# A Study on Urban Road Traffic Safety Based on Matter Element Analysis

**DOI:** 10.1155/2014/458483

**Published:** 2014-12-22

**Authors:** Qizhou Hu, Zhuping Zhou, Xu Sun

**Affiliations:** ^1^School of Automation, Nanjing University of Science and Technology, Nanjing 210094, China; ^2^Institute of Transportation Engineering, Tsinghua University, Beijing 100084, China

## Abstract

This paper examines a new evaluation of urban road traffic safety based on a matter element analysis, avoiding the difficulties found in other traffic safety evaluations. The issue of urban road traffic safety has been investigated through the matter element analysis theory. The chief aim of the present work is to investigate the features of urban road traffic safety. Emphasis was placed on the construction of a criterion function by which traffic safety achieved a hierarchical system of objectives to be evaluated. The matter element analysis theory was used to create the comprehensive appraisal model of urban road traffic safety. The technique was used to employ a newly developed and versatile matter element analysis algorithm. The matter element matrix solves the uncertainty and incompatibility of the evaluated factors used to assess urban road traffic safety. The application results showed the superiority of the evaluation model and a didactic example was included to illustrate the computational procedure.

## 1. Introduction

A major concern in the world today is the continued improvement of traffic safety. At present, urban road traffic safety has become a prominent social problem, causing large economic loss and human casualties. Considering all these issues, the study of urban road traffic safety has become more and more important [1]. The purpose of traffic safety evaluation of urban road is to make comparative satisfaction judgment, find out hidden safety trouble, take various corresponding measures, and reduce accident occurrence. Although much effort has been placed into improving these issues, an efficient and effective method has yet to be developed. The traffic safety system is extremely complex and many influential factors exist. Because of their uncertainty, it is difficult to quantify these factors and qualitative disposals are usually adopted. To evaluate urban road traffic safety, Wang et al. selected several factors of primary influence to evaluate the index, including road surface condition, traffic isolation, monitor establishment, congestion level, and accident ratio and tunnel establishment [2]. Taubman et al. implemented an evaluation by qualitative analysis [3]. The following flaws must be considered in this analysis: (1) the final appraisal cannot determine whether the urban road traffic safety condition as “good” or “bad”; (2) appraisal is based on an insufficient scientific nature, hindering its feasibility, and commensurability. There are no standard ways of assessing urban road traffic safety; therefore, scientific methods for such assessments are still needed.

During the past decade, the theory of matter element analysis has developed in numerous directions. Creating a quantitative appraisal model with quantitative evaluation provides the main study subject. However, there are many factors influencing urban road traffic safety. The actual conditions are quite complicated. It is very difficult to quantify the appraisal indices with specific functions and evaluate urban road traffic safety with a certain qualitative model [4]. Many evaluating processes take place in an environment in which the information is not precisely known [5]. Evaluation makers may have vague knowledge on the preference degrees of one alternative over another [6]. They cannot estimate their preference through exact numerical values, but through interval numbers. Based on the concept of interval numbers, a new method to solve the traffic safety problems is proposed in this paper. First, we define the concept of interval numbers and state the operational laws. Second, based on the concept of matter element, this paper offers a new method to solve the traffic safety problems through interval numbers. Finally, an example is given to demonstrate the feasibility and superiority of our proposed new method.

## 2. The Evaluation Index System for Urban Road Traffic Safety

The evaluation of urban road traffic safety is multifield and multisubject. The evaluation index selection must meet two standards: (1) the indices must enable the evaluation system to reflect the actual situation of urban road traffic safety completely and accurately and (2) the evaluation index system of urban road traffic safety must be as simple as possible.

### 2.1. The Traffic Safety Evaluation System of Urban Road

**Figure 1:**
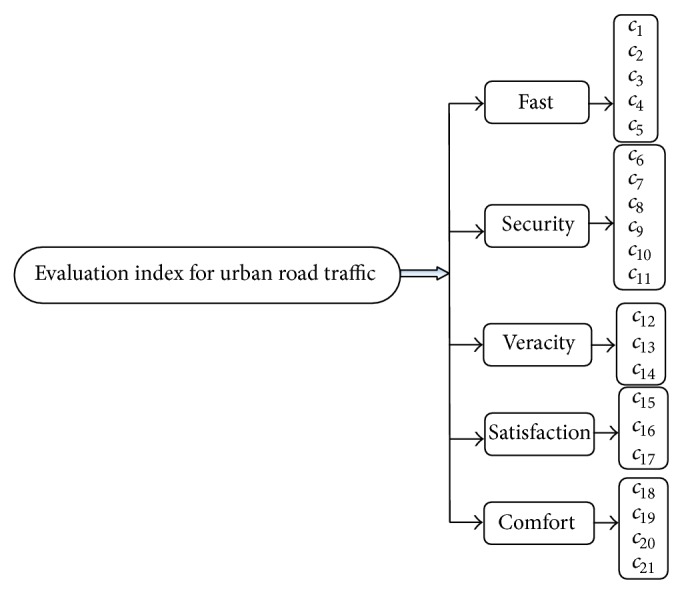
The evaluation system for urban road traffic safety.

In order to choose the evaluation indices correctly, we must make an accurate analysis on the traffic safety of urban road systems. Then the indices will reflect various influences objectively. Each evaluation index is selected by using the qualitative comparison method. For the convenience of the analysis of the computation, we obtain urban road safe evaluation target system through the generalized analysis of urban road traffic safety, as is shown in Figure 1.

*c*_1_ is the road network connectivity, *c*_2_ is the density of road network, *c*_3_ is chuckles level of joint, *c*_4_ is the speed of road network, *c*_5_ is road network volume, *c*_6_ is apparent distance, *c*_7_ is traffic segregation, *c*_8_ is protection facilities, *c*_9_ is lighting facilities, *c*_10_ is tunnel facilities, *c*_11_ is monitoring facilities, *c*_12_ is guide facilities, *c*_13_ is information system, *c*_14_ is weather forecast information facilities, *c*_15_ is pavement behavior, *c*_16_ is traffic accident rate, *c*_17_ is landscape along the line, *c*_18_ is mileage saturation rate, *c*_19_ is traffic congestion level, *c*_20_ is smooth-riding quality, and *c*_21_ is geographical features.

### 2.2. The Evaluation Indices of Urban Road Traffic Safety Quantified

Evaluation indices are classified into quantitative and qualitative indices. Each index has different meanings with different corresponding values [7]. In order to allow a common degree between each evaluation index of urban road traffic safety, we must carry standardized processing on the indices.

(1) The calculation method for the quantitative index of urban road traffic safety is as follows.

After the evaluation index is determined, we can then state the property value of the index of urban road traffic safety by either booking for statistical data, or deriving calculation.

(2) The calculation method for the qualitative index of urban road traffic safety is as follows.

It utilizes (an unclear) fuzzy function to complete the quantitative disposal for the qualitative index of urban road traffic safety. It imposes (an unclear) fuzzy transformation theory and maximal membership principle to set up quantitative evaluation conclusion without subjective factor [8]. Here it adopts (ambiguous) fuzzy language in mathematics that carry through standardization. It can be shown in Table 1.

### 2.3. The Grading Standards of the Evaluation Index of Urban Road Traffic Safety

Practically in evaluation making, the evaluation makers give the observed values of the index in the form of linguistic numbers, such as “bad, common, good, better, best,” or “low, common, high, higher, highest…” [9]. The main foundation of this paper is the application of rule-based decision-making. Since differences exist in the knowledge frame, experience level, status and individual preference for the evaluation makers, and incomplete, inadequate information is present in decision-making problems, so the observed values of the index given by the evaluation makers have a certain degree of grey area [10].

The factors mentioned above are divided into 5 grades (grade I, grade II, grade III, grade IV, and grade V) according to their influence on urban road traffic safety. Grade I indicates great safety influence in urban traffic; grade II indicates that safety influence exists on a small scale and has a large potential for urban traffic problems; grade III indicates safety influence of urban traffic exists in a large scale, but it still has some potentials; grade IV indicates that safety influence of urban traffic is near to the extremity; grade V indicates that safety influence of urban traffic is extreme. The evaluating standards can be seen in Table 2.

## 3. Matter Element Analysis Model of Urban Road Traffic Safety

In this section, basic terminologies and notations are introduced, necessarily to understand the subsequent results. The evaluation model, in absence of more precise insight, is based on various assumptions, some of which are simplified with respect to reality. At this stage, it is difficult to assess the validity and reliability of the evaluation model. It provides, however, a practical but founded and transparent method to address the problem of assessment of urban road traffic safety when only incomplete data are available, by enabling comparative analysis of urban road traffic safety with different nature. The evaluation model is also a valuable tool used to analyze the underlying mechanisms of traffic accident. The assumptions and resulting uncertainties concern the qualitative and quantitative analysis of the relationships between humans, vehicles, and road.

### 3.1. Basic Knowledge of Interval Numbers

Interval computation is a popular way to treat uncertainties in data measurements. Instead of exact values, we compute with real intervals, which are supposed to involve all gauging errors. An interval numbers is defined as $\left[\overset{-}{A}\right]=[a_{1},a_{2}]$, where 0 ≤ *a*_1_ ≤ *a*_2_ are fixed. *a*_1_ is the lower element in interval [*a*_1_, *a*_2_]. *a*_2_ is the upper elements in interval [*a*_1_, *a*_2_].

For the convenience of calculating interval numbers, some operational laws of interval numbers are introduced as follows [11]. Let $\left[\overset{-}{A}\right]=[a_{1},a_{2}]$ *and* $\left[\overset{-}{B}\right]=[b_{1},b_{2}]$ be interval numbers; then the basic operations on interval numbers are as follows:

1. [*a*_1_, *a*_2_] ± [*b*_1_, *b*_2_] = [*a*_1_ ± *b*_1_, *a*_2_ ± *b*_2_];
2. [*a*_1_, *a*_2_] · [*b*_1_, *b*_2_] = [*a*_1_ · *b*_1_, *a*_2_ · *b*_2_], [*a*_1_, *a*_2_] ÷ [*b*_1_, *b*_2_] = [*a*_1_/*b*_1_, *a*_2_/*b*_2_]  (*b*_1_ ≠ 0, *b*_2_ ≠ 0);
3. if *k* ≥ 0, then *k*[*a*_1_, *b*_1_] = [*ka*_1_, *kb*_1_], if *k* < 0, then *k*[*a*_1_, *b*_1_] = [*kb*_1_, *ka*_1_].

### 3.2. Basic Model of Matter Element

If the name, character, and value of a matter are provided, the three ordinal elements *R* = (*N*, *C*, *V*) are called a matter element [12, 13]. There are three basic elements of a matter element, for example, the matter's name *N*, the character *C*, and the value *V*. If a matter *N* has some characters and can be described by *n* characters *c*_1_, *c*_2_,…, *c*_*n*_ and its value *x*_1_, *x*_2_,…, *x*_*n*_, thus *R* = (*N*, *C*, *V*) is called an *n*-dimension matter element.

(1) Matter element matrix [14] is as follows:

(1) $$\begin{array}{c} R\left(\text{matter}\right)=\left[\begin{array}{ccc} N & c_{1} & x_{1}\\ & c_{2} & x_{2}\\ & \vdots & \vdots \\ & c_{n} & x_{n} \end{array}\right]. \end{array}$$

(2) Matter element matrix of classical field object is as follows.

Given *m* evaluating degrees as *N*_1_, *N*_2_,…, *N*_*m*_, the following matter element matrix of classical field object can be built as follows:

(2) $$\begin{array}{c} R_{i}\left(\text{classical}\right)=\left[\begin{array}{ccc} N_{i} & c_{1} & \left[{\overset{-}{X}}_{i1}\right]\\ & c_{2} & \left[{\overset{-}{X}}_{i2}\right]\\ & \vdots & \vdots \\ & c_{n} & \left[{\overset{-}{X}}_{in}\right] \end{array}\right], \end{array}$$

where *N*_*i*_ is standard object and *i* = 1,2,…, *m*, $\left[{\overset{-}{X}}_{ij}\right]=\left[a_{ij},b_{ij}\right]$ is the value range of *c*_*j*_  (*j* = 1,2,…, *n*) over standard object *N*_*i*_.

(3) Matter element matrix of section field object is as follows.

According to formula (2), we can construct its section filed

(3) $$\begin{array}{c} R_{i}\left(\text{section}\right)=\left[\begin{array}{ccc} N_{pi} & c_{1} & \left[{\overset{-}{X}}_{pi1}\right]\\ & c_{2} & \left[{\overset{-}{X}}_{pi2}\right]\\ & \vdots & \vdots \\ & c_{n} & \left[{\overset{-}{X}}_{pin}\right] \end{array}\right], \end{array}$$

where *N*_*pi*_ is section object, $\left[{\overset{-}{X}}_{pij}\right]=\left[a_{pij},b_{pij}\right]$ is the interval range of the measured value of the character *C* about section field object, and $\left[{\overset{-}{X}}_{pij}\right]\subset \left[{\overset{-}{X}}_{ij}\right]\;\;(j=1,2,\ldots ,n)$.

### 3.3. The Comprehensive Appraisal Model of Urban Road Traffic Safety

Urban road traffic safety is a crucial subject. Determining the safety levels may help prevent future traffic accidents in the city. But it is not an easy task. Many parameters have considerable effects on the phenomenon. Determining safety levels has a degree of uncertainty. This study deals with determination of city safety levels using order matter element. The analysis approach considers accident types and effective factors on accident occurrence. Geometrical and physical conditions, traffic volumes, average speeds, and average accident rates at around traffic safety are considered as effective factors in city. Matter element matrix is calculated using these parameters. Validation of matter element analysis approach is tested by truth-value methods, and encouraging results are obtained.

*Step 1 (matter element matrix of urban road traffic safety)*. For the estimated object *P*_*i*_, the prediction result of urban road traffic safety can be represented by the following matter element matrix of urban road traffic safety:

(4) $$\begin{array}{c} R\left(P_{i}\right)=\left[\begin{array}{ccc} P_{i} & c_{1} & x_{i1}\\ & c_{2} & x_{i2}\\ & \vdots & \vdots \\ & c_{n} & x_{in} \end{array}\right]. \end{array}$$

*Step 2 (the distance calculation of matrix for urban road traffic safety).*

**Definition 1 .**

If $\left[{\overset{-}{X}}_{ij}\right]=\left[a_{ij},b_{ij}\right]$, then the relating coefficient of urban road traffic safety is

(5) $$\begin{array}{c} \rho \left(x_{ij},\left[{\overset{-}{X}}_{ij}\right]\right)=\left|x_{ij}-0.5\left(a_{ij}+b_{ij}\right)\right|-0.5\left(b_{ij}-a_{ij}\right),\\ \rho \left(x_{ij},\left[{\overset{-}{X}}_{pj}\right]\right)=\left|x_{ij}-0.5\left(a_{pj}+b_{pj}\right)\right|-0.5\left(b_{pj}-a_{pj}\right), \end{array}$$

where *i* = 1,2,…, *m* and *j* = 1,2,…, *n*.

*Step 3 (relation function about urban road traffic safety).*

**Definition 2 .**

If $\left[{\overset{-}{X}}_{ij}\right]=\left[a_{ij},b_{ij}\right]$, then the relating function of urban road traffic safety is

(6) $$\begin{array}{c} K\left(x_{ij}\right)=\left\{\begin{array}{cc} -\rho \left(x_{ij},\left[{\overset{-}{X}}_{ij}\right]\right)\cdot {\left|\left[{\overset{-}{X}}_{ij}\right]\right|}^{-1}, & x_{ij}\in \left[{\overset{-}{X}}_{ij}\right]\\ \rho \left(x_{ij},\left[{\overset{-}{X}}_{ij}\right]\right)\cdot {\left[\rho \left(x_{ij},\left[{\overset{-}{X}}_{pj}\right]\right)-\rho \left(x_{ij},\left[{\overset{-}{X}}_{ij}\right]\right)\right]}^{-1}, & x_{ij}\notin \left[{\overset{-}{X}}_{ij}\right], \end{array}\right. \end{array}$$

where *i* = 1,2,…, *m* and *j* = 1,2,…, *n*.

*Step 4 (entropy-weight coefficient of the index system of urban road traffic safety)*. Traditional AHP [15] focuses on the knowledge and experience of experts as well as their wishes and preferences of decision-making thus unable to overcome the subjective and arbitrary mistakes. Entropy-weight can fully exploit the information implied in the original data itself, but it lacks the knowledge and experience from experts and decision-makers. So together, the two methods complement each other. The original information contains quality and quantity, which make it possible to weigh the information in the decision work. Let us denote by

(7) $$\begin{array}{c} h_{ij}=-\frac{1}{\ln m}\overset{m}{\underset{i=1}{\sum }}x_{ij}\ln x_{ij}, \end{array}$$

where *i* = 1,2,…, *m* and *j* = 1,2,…, *n* and set *x*_*ij*_ln⁡*x*_*ij*_ = 0 while *x*_*ij*_ = 0.

Then, the weight value of urban road traffic safety can be calculated by (8)

(8) $$\begin{array}{c} w_{ij}=\left(1-h_{ij}\right)\cdot {\left(m-\overset{m}{\underset{j=1}{\sum }}h_{ij}\right)}^{-1}. \end{array}$$

*Step 5 (the relating degree of urban road traffic safety)*. According to the weight of the appraisal index value *w*_*ij*_ and the relation function *K*(*x*_*ij*_), the comprehensive appraisal value of urban road traffic safety is obtained as follows:

(9) $$\begin{array}{c} K\left(P_{i}\right)=\overset{n}{\underset{j=1}{\sum }}w_{ij}K\left(x_{ij}\right)\;\left(i=1,2,\ldots ,m\right). \end{array}$$

Then *K*(*P*_*i*_) is defined as the relating degree of the evaluated matter *P*_*i*_ with regard to *i*'s grade. According to the principle of the maximum dependence, the relating function value *K*(*P*_*i*_) is maximized:

(10) $$\begin{array}{c} K_{i0}=\underset{1\leq i\leq m}{\max}\left\{K\left(P_{i}\right)\right\}. \end{array}$$

Hence, the estimating matter *P*_*i*_ should belong to the *i*_0_'s grade by CAI Wen.

Appraisal set is a set which we get appraisal conclusion for appraisal target [16, 17]. The traffic safety comprehensive appraisal grade can be obtained in appraisal set *F*. Appraisal set *F* = {the first grade (i.e., “Excellent”), the second grade (i.e., “Good”), the third grade (i.e., “Medium”), the fourth grade (i.e., “General”), the fifth grade (i.e., “Poor”)}. 

*Step 6 (the appraisal standardization of urban road traffic safety)*. Based on the comprehensive appraisal value *K*(*P*_*i*_) and the appraisal grade standard of urban road traffic safety, the comprehensive appraisal grade of urban road traffic safety is analyzed and hence obtained. This value quantitatively reflects urban road traffic safety “good” or “bad” and provides the scientific basis of decision-making for urban road construction. The greater the comprehensive appraisal value *K*(*P*_*i*_), the better the urban road traffic safety in the monitoring period. In counterpart, the smaller the comprehensive appraisal value *K*(*P*_*i*_), the worse the urban road traffic safety in the monitoring period.

## 4. Study on the Measures of Urban Road Traffic Safety

It is necessary and desired to solve the problems of the urban road traffic safety by using comprehensive management knowledge from all sides. In the light of features of the urban road traffic safety, the analysis results achieved in the paper suggest the following: (i) on one hand, it is essential to improve the supervision and control measures of traffic safety. Urban road has been installed with advanced and modernized facilities, among which traffic control system is the key approach for urban road traffic management [18]. The traffic control system meets the needs of traffic control, monitoring, accessibility, and communication. Under the circumstances of adverse weather and traffic jams, the system can lead drivers to the safe drive and provide traffic supervision and a control system. (ii) On the other hand, vigorous measures are adopted to develop intelligent transportation systems. Intelligent systems are the front topics in the field of transportation all over the world. Intelligent transportation systems are capable of improving traffic capacity and safety of existing networks. They represent the development orientation of traffic science [19]. When the traffic science and technology have great breakthroughs and developments, intelligent transportation technologies can decrease traffic congestion and traffic accident, heighten the labor productivity, intensify the international competition, and increase new industry in future. By means of high-tech, computer science, and communication technology, people can improve the systematic engineering of electromechanical devices for communication, charges, and supervision and monitoring which can make intelligent urban road grow. Developing intelligent urban road can resolve traffic jams, ensure driving safety, and improve service quality. In a word, it can represent the characteristics of modernized urban road [20].

## 5. Application Analyses

**Figure 2:**
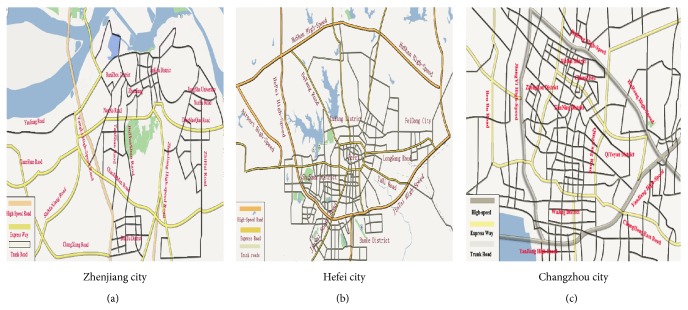
The map for three cities.

In order to carry out rational appraisal to urban road traffic safety in three cities, for example, Zhenjiang city, Hefei city, and Changzhou city, in China are shown in Figure 2, urban road on-the-spot from June to July in 2010 was surveyed, and a lot of effective data about the traffic safety were obtained which could quantitatively and comprehensively evaluate the road safety of the three cities. The specific values *x*_*ij*_ are shown in Table 3.

*Step 1*. Determine matter element matrix *R* and section filed *R*_*i*_ of urban road traffic safety.

According to Table 1, take value region corresponding to the evaluating criterion of grades I–V as the sutra filed *R*_*i*_.

(1) For the first city (i.e., “Zhenjiang city in China”)

(11) $$\begin{array}{c} R_{1}\left(\text{matter}\right)=\left[\begin{array}{ccc} N & c_{1} & 2.81\\ & c_{2} & 6.72\\ & c_{3} & 0.71\\ & c_{4} & 36.7\\ & c_{5} & 3.80\\ & c_{6} & 6.74\\ & c_{7} & 0.62\\ & c_{8} & 0.65\\ & c_{9} & 0.77\\ & c_{10} & 0.68\\ & c_{11} & 0.61\\ & c_{12} & 0.78\\ & c_{13} & 0.61\\ & c_{14} & 0.73\\ & c_{15} & 0.68\\ & c_{16} & 0.035\\ & c_{17} & 0.68\\ & c_{18} & 0.75\\ & c_{19} & 0.57\\ & c_{20} & 0.68\\ & c_{21} & 0.78 \end{array}\right],\\ R_{1}\left(\text{classical}\right)=\left[\begin{array}{ccc} N_{1} & c_{1} & \left[2.5,3.0\right]\\ & c_{2} & \left[6.0,8.0\right]\\ & c_{3} & \left[0.7,0.9\right]\\ & c_{4} & \left[30,40\right]\\ & c_{5} & \left[3.5,4.5\right]\\ & c_{6} & \left[5.0,10.0\right]\\ & c_{7} & \left[0.6,0.7\right]\\ & c_{8} & \left[0.6,0.7\right]\\ & c_{9} & \left[0.7,0.8\right]\\ & c_{10} & \left[0.6,0.7\right]\\ & c_{11} & \left[0.6,0.7\right]\\ & c_{12} & \left[0.7,0.8\right]\\ & c_{13} & \left[0.6,0.7\right]\\ & c_{14} & \left[0.7,0.8\right]\\ & c_{15} & \left[0.6,0.7\right]\\ & c_{16} & \left[0.03,0.04\right]\\ & c_{17} & \left[0.6,0.7\right]\\ & c_{18} & \left[0.7,0.8\right]\\ & c_{19} & \left[0.5,0.6\right]\\ & c_{20} & \left[0.6,0.8\right]\\ & c_{21} & \left[0.7,0.8\right] \end{array}\right]. \end{array}$$

(2) For the second city (i.e., “Hefei city in China”)

(12) $$\begin{array}{c} R_{2}\left(\text{matter}\right)=\left[\begin{array}{ccc} N & c_{1} & 3.12\\ & c_{2} & 6.91\\ & c_{3} & 0.54\\ & c_{4} & 53.7\\ & c_{5} & 3.17\\ & c_{6} & 6.65\\ & c_{7} & 0.71\\ & c_{8} & 0.67\\ & c_{9} & 0.61\\ & c_{10} & 0.55\\ & c_{11} & 0.63\\ & c_{12} & 0.58\\ & c_{13} & 0.53\\ & c_{14} & 0.71\\ & c_{15} & 0.54\\ & c_{16} & 0.037\\ & c_{17} & 0.61\\ & c_{18} & 0.73\\ & c_{19} & 0.68\\ & c_{20} & 0.73\\ & c_{21} & 0.69 \end{array}\right],\\ R_{2}\left(\text{classical}\right)=\left[\begin{array}{ccc} N_{2} & c_{1} & \left[3.0,3.5\right]\\ & c_{2} & \left[6.0,8.0\right]\\ & c_{3} & \left[0.5,0.6\right]\\ & c_{4} & \left[40,100\right]\\ & c_{5} & \left[3.5,4.5\right]\\ & c_{6} & \left[5.0,10.0\right]\\ & c_{7} & \left[0.7,0.8\right]\\ & c_{8} & \left[0.6,0.7\right]\\ & c_{9} & \left[0.6,0.7\right]\\ & c_{10} & \left[0.0,0.6\right]\\ & c_{11} & \left[0.0,0.6\right]\\ & c_{12} & \left[0.0,0.6\right]\\ & c_{13} & \left[0.0,0.6\right]\\ & c_{14} & \left[0.7,0.8\right]\\ & c_{15} & \left[0.0,0.6\right]\\ & c_{16} & \left[0.03,0.04\right]\\ & c_{17} & \left[0.6,0.7\right]\\ & c_{18} & \left[0.7,0.8\right]\\ & c_{19} & \left[0.6,0.7\right]\\ & c_{20} & \left[0.6,0.8\right]\\ & c_{21} & \left[0.6,0.7\right] \end{array}\right]. \end{array}$$

(3) For the third city (i.e., “Changzhou city in China”)

(13) $$\begin{array}{c} R_{3}\left(\text{matter}\right)=\left[\begin{array}{ccc} N & c_{1} & 2.13\\ & c_{2} & 6.21\\ & c_{3} & 0.68\\ & c_{4} & 39.8\\ & c_{5} & 3.67\\ & c_{6} & 6.71\\ & c_{7} & 0.69\\ & c_{8} & 0.71\\ & c_{9} & 0.67\\ & c_{10} & 0.58\\ & c_{11} & 0.73\\ & c_{12} & 0.68\\ & c_{13} & 0.63\\ & c_{14} & 0.58\\ & c_{15} & 0.64\\ & c_{16} & 0.043\\ & c_{17} & 0.71\\ & c_{18} & 0.63\\ & c_{19} & 0.58\\ & c_{20} & 0.63\\ & c_{21} & 0.71 \end{array}\right],\\ R_{3}\left(\text{classical}\right)=\left[\begin{array}{ccc} N_{3} & c_{1} & \left[0.0,2.5\right]\\ & c_{2} & \left[6.0,8.0\right]\\ & c_{3} & \left[0.6,0.7\right]\\ & c_{4} & \left[30,40\right]\\ & c_{5} & \left[3.5,4.5\right]\\ & c_{6} & \left[5.0,10.0\right]\\ & c_{7} & \left[0.6,0.7\right]\\ & c_{8} & \left[0.7,0.8\right]\\ & c_{9} & \left[0.6,0.7\right]\\ & c_{10} & \left[0.0,0.6\right]\\ & c_{11} & \left[0.7,0.8\right]\\ & c_{12} & \left[0.6,0.7\right]\\ & c_{13} & \left[0.6,0.7\right]\\ & c_{14} & \left[0.0,0.6\right]\\ & c_{15} & \left[0.6,0.7\right]\\ & c_{16} & \left[0.04,0.05\right]\\ & c_{17} & \left[0.7,0.8\right]\\ & c_{18} & \left[0.6,0.7\right]\\ & c_{19} & \left[0.5,0.6\right]\\ & c_{20} & \left[0.6,0.8\right]\\ & c_{21} & \left[0.7,0.8\right] \end{array}\right]. \end{array}$$

*Step 2*. Determine the section field *R*_*p*_ of urban road traffic safety:

(14) $$\begin{array}{c} R_{p}\left(\text{section}\right)=\left[\begin{array}{ccc} N_{p} & c_{1} & \left[0.0,1.0\right]\\ & c_{2} & \left[0.0,10.0\right]\\ & c_{3} & \left[0.0,1.0\right]\\ & c_{4} & \left[0.0,1.0\right]\\ & c_{5} & \left[0.0,10.0\right]\\ & c_{6} & \left[0.0,1.0\right]\\ & c_{7} & \left[0.0,1.0\right]\\ & c_{8} & \left[0.0,1.0\right]\\ & c_{9} & \left[0.0,1.0\right]\\ & c_{10} & \left[0.0,1.0\right]\\ & c_{11} & \left[0.0,1.0\right]\\ & c_{12} & \left[0.0,1.0\right]\\ & c_{13} & \left[0.0,1.0\right]\\ & c_{14} & \left[0.0,1.0\right]\\ & c_{15} & \left[0.0,1.0\right]\\ & c_{16} & \left[0.0,1.0\right]\\ & c_{17} & \left[0.0,1.0\right]\\ & c_{18} & \left[0.0,1.0\right]\\ & c_{19} & \left[0.0,1.0\right]\\ & c_{20} & \left[0.0,1.0\right]\\ & c_{21} & \left[0.0,1.0\right] \end{array}\right]. \end{array}$$

Get the section filed *R*_*p*_ according to the value region of the evaluating element in the Tables 1 and 3.

*Step 3*. Obtain the relation function values *K*(*x*_*ij*_) of the evaluation indices by formula (6) in Table 3. 

*Step 4*. Obtain the weight values of the evaluation indices by formula (8) in Table 3. 

*Step 5*. Obtain the synthetic relating degree and the result of evaluation.

Calculated by (5) and (6), the synthetic relating degree of the urban road traffic safety in three cities is shown in Table 4.

The evaluation results in Table 4 show that the road traffic safety of the third city, Changzhou city, belongs to Level 2, and that city, however, is basically sustainable developing. Urban road traffic safety of the second city, Hefei city, belongs to Level 4, meaning that the sustainable development is close to the extremity. It is important for the second city, Changzhou city, to find new methods and advanced management technologies to develop urban traffic system and hence increase sustainable development capacity. In the same manner, the first city, Zhenjiang city, belongs to Level 3. Therefore it is concluded that road traffic safety is basically rational and basically sustainable developing in one city. However, we should raise sustainable development level for road traffic safety in one city, accelerate intelligence and information for urban traffic system, and promote coordinated development in entire city in future plan.

## 6. Conclusions

In this paper the urban road traffic safety evaluation problems have been studied. An objective-evaluating model has been developed to determine the grade of the safety of the city, as well as a practical approach to rank the safety of the city. Based on the concept of matter element, this paper offers a new method to solve the traffic safety problems through interval numbers. Emphasis was placed on the construction of a criterion function by which traffic safety achieved a hierarchical system of objectives to be evaluated. The matter element matrix solves the uncertainty and incompatibility of the evaluated factors used to assess urban road traffic safety. The application results showed that the new model is more reasonable and persuasive.

Conclusions and future work are summarized as well. The proposed method provides a relatively objective basis for the short-term and long-term planning of the protection of urban resources and creates a foundation for the traffic management and urban traffic sustainable development. Moreover, it provides a scientific basis for the policy decision of urban traffic construction and a train of new thought for evaluated method in this field. It is necessary for urban road to adopt advanced technologies to increase the safety.

## Figures and Tables

**Table 1:** Quantification of the evaluation index.

Criterion	Excellent	Good	Medium	General	Poor
Evaluation interval	[0.9, 1]	[0.8, 0.9]	[0.7, 0.8]	[0.6, 0.7]	[0.0, 0.6]

**Table 2:** The grading standards of the evaluating index.

Evaluation index	Grade I	Grade II	Grade III	Grade IV	Grade V
	Excellent	Good	Medium	General	Poor
*c*_1_	0~0.2	0.2~0.4	0.4~0.6	0.6~0.8	>0.8
*c*_2_ (km/km^2^)	0~2.5	2.5~3.0	3.0~3.5	3.5~4.5	>4.5
*c*_3_	0~0.5	0.5~0.6	0.6~0.7	0.7~0.9	>0.9
*c*_4_ (km/h)	0~10	10~20	20~30	30~40	>40
*c*_5_ (standard units/km)	>5.5	4.5~5.5	3.5~4.5	2.5~3.5	0~2.5
*c*_6_ (m)	0~5	5~10	10~15	15~20	>20
*c*_7_	0~0.6	0.6~0.7	0.7~0.8	0.8~0.9	>0.9
*c*_8_	0~0.6	0.6~0.7	0.7~0.8	0.8~0.9	>0.9
*c*_9_	0~0.6	0.6~0.7	0.7~0.8	0.8~0.9	>0.9
*c*_10_	0~0.6	0.6~0.7	0.7~0.8	0.8~0.9	>0.9
*c*_11_	0~0.6	0.6~0.7	0.7~0.8	0.8~0.9	>0.9
*c*_12_	0~0.6	0.6~0.7	0.7~0.8	0.8~0.9	>0.9
*c*_13_	0~0.6	0.6~0.7	0.7~0.8	0.8~0.9	>0.9
*c*_14_	0~0.6	0.6~0.7	0.7~0.8	0.8~0.9	>0.9
*c*_15_	0~0.6	0.6~0.7	0.7~0.8	0.8~0.9	>0.9
*c*_16_ (%)	>0.05	0.04~0.05	0.03~0.04	0.02~0.03	0~0.02
*c*_17_	0~0.6	0.6~0.7	0.7~0.8	0.8~0.9	>0.9
*c*_18_ (%)	>0.8	0.7~0.8	0.6~0.7	0.5~0.6	0~0.5
*c*_19_	>0.8	0.7~0.8	0.6~0.7	0.5~0.6	0~0.5
*c*_20_	0~0.2	0.2~0.4	0.4~0.6	0.6~0.8	>0.8
*c*_21_	0~0.6	0.6~0.7	0.7~0.8	0.8~0.9	>0.9

**Table 3:** The observed values of evaluation indexes.

Index	*x*_*ij*_			*w*_*ij*_			*K*(*x*_*ij*_)		
	Zhenjiang	Hefei	Changzhou	Zhenjiang	Hefei	Changzhou	Zhenjiang	Hefei	Changzhou
	The first city	The second city	The third city	The first city	The second city	The third city	The first city	The second city	The third city
*c*_1_	2.81	3.12	2.13	0.029	0.051	0.043	0.082	0.107	0.076
*c*_2_	6.72	6.91	6.21	0.051	0.039	0.046	0.138	0.117	0.143
*c*_3_	0.71	0.54	0.68	0.043	0.067	0.063	0.235	0.198	0.212
*c*_4_	36.7	53.7	39.8	0.067	0.043	0.047	0.156	0.136	0.141
*c*_5_	3.80	3.17	3.67	0.024	0.026	0.037	0.098	0.089	0.101
*c*_6_	6.74	6.65	6.71	0.026	0.034	0.023	0.191	0.198	0.203
*c*_7_	0.62	0.71	0.69	0.059	0.061	0.058	0.106	0.099	0.097
*c*_8_	0.65	0.67	0.71	0.061	0.059	0.062	0.096	0.101	0.103
*c*_9_	0.77	0.61	0.67	0.023	0.047	0.046	0.103	0.174	0.169
*c*_10_	0.68	0.55	0.58	0.047	0.033	0.034	0.145	0.147	0.138
*c*_11_	0.61	0.63	0.73	0.022	0.038	0.041	0.061	0.087	0.092
*c*_12_	0.78	0.58	0.68	0.028	0.032	0.029	0.105	0.132	0.123
*c*_13_	0.61	0.53	0.63	0.031	0.049	0.038	0.176	0.185	0.134
*c*_14_	0.73	0.71	0.58	0.049	0.031	0.042	0.136	0.158	0.168
*c*_15_	0.68	0.54	0.64	0.036	0.044	0.037	0.195	0.201	0.198
*c*_16_	0.035	0.037	0.043	0.034	0.036	0.034	0.168	0.173	0.157
*c*_17_	0.68	0.61	0.71	0.062	0.042	0.049	0.035	0.047	0.051
*c*_18_	0.75	0.73	0.63	0.063	0.052	0.061	0.065	0.068	0.059
*c*_19_	0.57	0.68	0.58	0.058	0.076	0.066	0.126	0.117	0.131
*c*_20_	0.68	0.73	0.63	0.076	0.058	0.048	0.167	0.154	0.144
*c*_21_	0.78	0.69	0.71	0.081	0.052	0.072	0.128	0.137	0.123

**Table 4:** The evaluating result and the synthetic relating degree in each city.

Name		The grade of urban road traffic safety					The evaluation result
		Very safe	Safe	Approximately safe	Unsafe	Very safe	
		Level 1	Level 2	Level 3	Level 4	Level 5	
The first city	Zhenjiang	0.1127	0.2732	0.3013	0.1889	0.2229	Level 3
The second city	Hefei	0.1237	0.1721	0.2136	0.2681	0.2335	Level 4
The third city	Changzhou	0.1711	0.1882	0.2027	0.2032	0.2362	Level 2
